# Detection of recombinant breakpoint in the genome of human enterovirus E11 strain associated with a fatal nosocomial outbreak

**DOI:** 10.1186/s12985-022-01821-2

**Published:** 2022-06-03

**Authors:** Martina Rueca, Simone Lanini, Emanuela Giombini, Francesco Messina, Concetta Castilletti, Giuseppe Ippolito, Maria Rosaria Capobianchi, Maria Beatrice Valli

**Affiliations:** National Institute for Infectious Diseases L. Spallanzani IRCCS, Rome, Italy

**Keywords:** Enterovirus, Recombination, Phylogeny

## Abstract

**Background:**

The aim of this study was to characterize the genome of a recombinant Enterovirus associated with severe and fatal nosocomial infection; it was typed as Echovirus 11 (E-11) according to the VP1 gene. Enterovirus infection is generally asymptomatic and self-limited, but occasionally it may progress to a more severe clinical manifestation, as in the case described here. Recombination plays a crucial role in the evolution of Enteroviruses (EVs) and has been recognized as the main driving force behind the emergence of epidemic strains associated with severe infection. Therefore, it is of utmost importance to monitor the circulation of recombinant strains for surveillance purposes.

**Methods:**

Enterovirus-RNA was detected in the serum and liver biopsy of patients involved in the nosocomial cluster by commercial One-Step qRT-PCR method and the Enterovirus strains were isolated in vitro*.* The EVs typing was determined by analyzing the partial-length of the 5′UTR and VP1 sequences with the web-based open-access Enterovirus Genotyping Tool Version 0.1. The amplicons targeting 5′UTR, VP1 and overlapping fragments of the entire genome were sequenced with the Sanger method. Phylogenetic analysis was performed comparing the VP1 and the full-genome sequences of our strains against an appropriate reference set of Enterovirus prototypes of the Picornaviridae genera and species retrieved from the Enterovirus Genotyping Tool. Recombination analysis was performed using RDP4 software.

**Results:**

The Neighbor-Joining tree of the VP1 gene revealed that the 4 patients were infected with an identical molecular variant of Echovirus 11 (E-11). While the phylogenetic and the RDP4 analysis of the full-genome sequences provided evidence that it was a chimeric strain between an E-11 and a Coxsackievirus B (CV-B).

**Conclusions:**

The chimeric structure of the E-11 genome might have contributed to the severe infection and epidemic feature of the strain, but further biological characterizations are needed. The evidence reported in this study, highlights the limit of typing techniques based on the VP1 gene, as they fail to identify the emergence of recombinant strains with potentially more pathogenic or epidemic properties, thus providing only partial information on the epidemiology and pathogenesis of Enteroviruses.

**Supplementary Information:**

The online version contains supplementary material available at 10.1186/s12985-022-01821-2.

## Background

Enteroviruses are a large genus of non-enveloped single stranded positive-sense RNA viruses of the *Picornaviridae* family. The genus consists of 15 species, which contain over 100 different serotypes, only eight of which (Enterovirus A–D and Rhinovirus A to C) have been recognized to infect humans [1].

EVs are the most common worldwide circulating viruses and are characterized by a great phenotypic variability. Indeed, they are responsible for a wide spectrum of clinical illnesses, ranging from mild respiratory or gastro-intestinal symptoms to more severe clinical outcomes, such as acute and chronic cardiac disease, hepatitis, meningitis and encephalitis. The transmission of the infection is generally through the fecal–oral route, but may also occurs via respiratory droplets.

The genome is a single stranded positive-sense RNA of 7,100 to 8,500 nucleotides long with a poly(A) tail at its 3′ end. It contains a single Open Reading Frame (ORF) encoding for a unique polyprotein, which is cleaved by virus-encoded proteases (2A, 3C and 3CD) to yield four capsid proteins (VP1-4) and seven non-structural proteins as the proteases and the RNA-dependent-RNA polymerase. The viral cycle is entirely cytoplasmatic, and the RNA genome replication proceeds via the synthesis of negative-sense RNA copy of the viral genome.

The ORF is flanked by two untranslated regions (UTR) at 5′ and 3′ respectively that play an important functional role in viral biology [2].

The 5′UTR is approximately 750 nucleotides long and contains two major domains: a cloverleaf motif essential for positive-strand synthesis (90 nt), and an internal ribosome entry site (IRES) which is involved in cap-independent translation initiation. Studies on the pathogenesis of the poliovirus have emphasized a role for the 5′UTR in the neuro-virulence phenotypes [3].

In contrast, the 3′UTR is a much shorter sequence (70–100 nucleotides) compared to the 5′UTR and comparatively little studied, although it maps in close proximity to the initiation site of negative-strand synthesis and might probably play a crucial role in the replication process [4].

As all RNA viruses, the genome of the Enteroviruses can evolve at high mutation rates and all members of the genus are known to be among the most rapidly changing viruses. According to previous studies, the Poliovirus and the Non-Polio Enterovirus VP1 gene accumulate 5–15 × 10^−3^ substitution per site per year [5]. The evolution of EVs is deeply influenced by two main factors: an RNA polymerase without a proof-reading activity and the high probability of EVs genome to undergo a recombination event. The latter plays a crucial role in EVs evolution and specifically intertypic recombination has been recognized as the major driving force behind the epidemic circulation profile of E-9, E-11 and E-30 [6–8]. In addition, it is likely that both mechanisms may account for the wide genetic and antigenic heterogeneity of the genus as well as strongly contributing to the emergence of new variants with a potentially greater clinical burden and epidemic features.

In the past decade, two well-known serotypes often associated to sporadic and mild infections, were considered emerging pathogens having caused epidemics of severe infection with an upsurge in hospitalizations. Enterovirus 71 (species A), commonly implicated in Hand, Foot and Mouth Disease (HFMD), has been recently associated with an increased risk of neurological disease [9] and the Enterovirus D68 (species D) has been reported as a significant respiratory pathogen associated with an apparent increase in cases of acute flaccid paralysis (AFP) [10]. Since it caused an outbreak in 2014 in the USA, it has started to spread globally and cases are increasing as recently reported by UK (ECDC) and United States (CDC) [11]. Furthermore, given the rapid evolution of the Genus, new serotypes are continuously being identified, such as EV-B80, EV-B83 and EV-B93 reported in China in the latest years [12–14].

In July 2013, a 73-years-old woman diagnosed with relapsing non-Hodgkin lymphoma (NHL) and psoriatic arthritis, died with cholestatic hepatitis and underwent autopsy (Pt_0 index case). After approximately a week from the autopsy, three (Pt_1, Pt_2 and Pt_3) of the six persons who took part in the post-mortem procedures developed acute hepatitis and one of them eventually died from acute liver failure. All patients resulted positive to Enterovirus RNA and the sequence typing of the VP1 gene revealed that it was an Echovirus 11 (E-11).

In this paper, we analyzed the whole genome sequence of the E-11 strain involved in this small nosocomial outbreak, to explore the genetic factors underlying increased virulence.

## Methods

### Study participants and sample collection

Serum and biopsy samples were collected from four patients involved in the nosocomial outbreak: Pt0 (the index case), Pt1, Pt2 and Pt3 (Table 1). As Pt1 was the first one to develop severe symptoms and acute hepatitis after the autopsy, a liver biopsy was extracted after mechanical homogenization and underwent EBV, CMV, HSV1-2, HBV, and HHV8 DNA and Enterovirus, Flavivirus, Arenavirus and Hantavirus RNA amplification with commercial and in house real-time PCR or RT-PCR. Only the Enterovirus test resulted positive with RealTime RT-PCR (RQ ENTEROVIRUS, AB analitica, Padova, Italy). The serum of Pt2 and Pt3, and the embedded paraffin liver biopsy were analyzed for Enterovirus diagnosis with all specimens resulting positive.

**Table 1:** Legend of collected and analyzed samples

Patient	Sample type
Pt0	Liver biopsy (paraffine embedded)
Pt1	Liver biopsy
	Serum
Pt2	Serum
Pt3	Serum

### Isolation

Vero E6 cells (ATCC® number CRL-1586™) were maintained in Modified Eagle Medium (MEM) supplemented with 10% heat-inactivated fetal calf serum (FCS) at 37 °C in a humidified atmosphere of 5% CO_2_. For viral culture assay, Vero E6 cells were infected with clinical samples of infected patients. Specifically, three Enterovirus isolates were isolated from the native liver biopsy of patient 1 (Iso_pt1_L) and from the serum of patient 2 and patient 3 (Iso_pt2_S and Iso_pt3_S respectively).

### Extraction, amplification and sequencing

The Viral RNA was extracted from cell supernatants and clinical samples using RNeasy mini kit (QIAGEN), RNA from the embedded paraffin liver was extracted performing a pre-treatment with EZ1® RNA Tissue Mini Kit (Qiagen; Hilden, Germany) according to the manufacturer’s instructions. The RNA amplification of partial VP1 and UTR gene and full genome sequencing was performed using Qiagen OneStep RT-PCR kit (QIAGEN) according to the manufacturer’s instructions. The amplicons were sequenced using ABI prism 3130 × 1 GENETIC ANALYZER DNA SEQUENCER.

### Genotyping

**Fig. 1:**
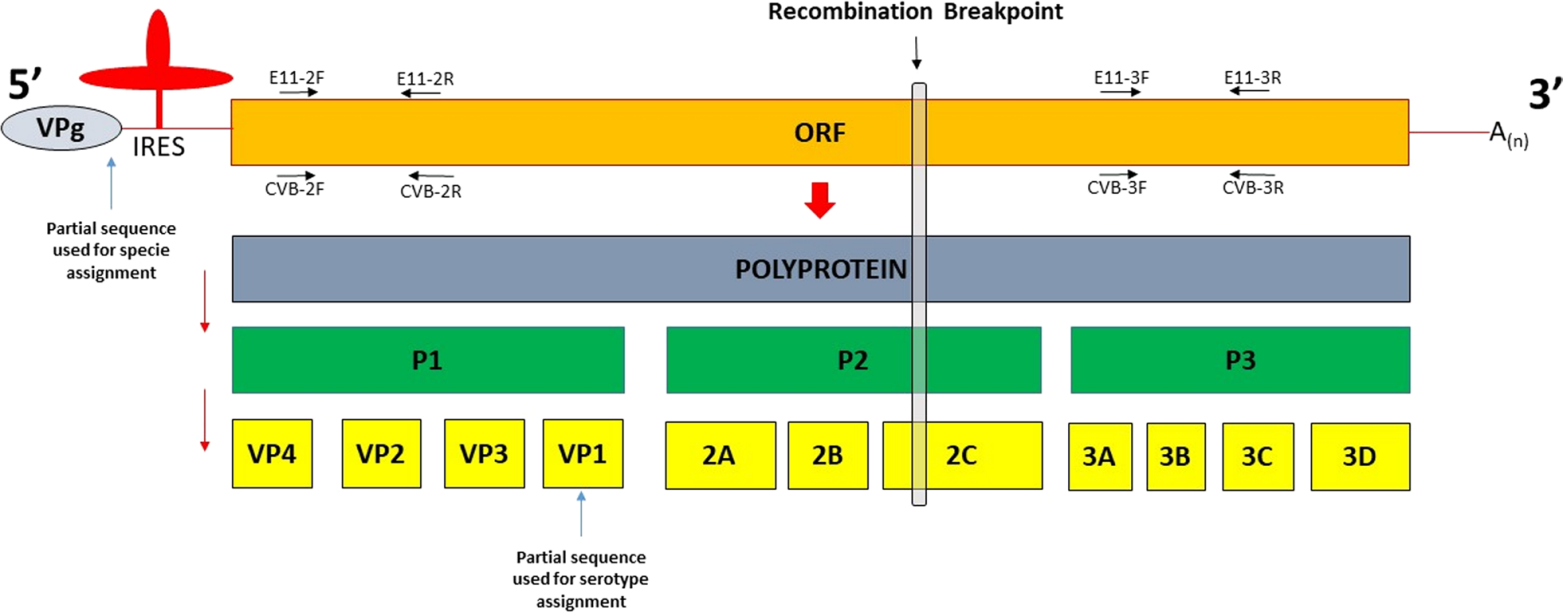
Schematic representation of enterovirus genome and the recombination breakpoint site. The blue arrows indicate the regions used for typing of EVs (5′UTR used for specie assignment and VP1 used for serotype assignment); primers used for Specific RT-PCR for E-11 and CV-B are indicated by black arrows

The typing was performed using the Enterovirus Genotyping Tool (https://www.rivm.nl/mpf/typingtool/enterovirus/) [15]. Species assignment was obtained on the basis of the partial sequence of 5′ Untranslated Region (UTR) while VP1 partial sequence was used for serotype assignment (Fig. 1). The 5′UTR partial region was amplified using primers described by Nicholson et al. [16] while amplification of a portion of VP1 gene was obtained using primers AN88-AN89, by Nix et al. [17].

### Full-length genome sequencing

The RNA genome of the three Enterovirus isolates (Iso_pt1_L, Iso_pt2_S and Iso_pt3_S) and of the one derived from the native liver biopsy of patient 1 (Pt1_L) were amplified in 23 overlapping reverse transcriptase-PCR amplicons, using One-Step RT-PCR Kit (QIAGEN; Hilden, Germany) and sequenced with the Sanger method (oligonucleotide sequences are supplied in Additional file 1). The nucleotide sequences were edited and aligned by using Clustal W [19] and BioEdit v 7.0.5.3 software, to reconstruct the full-length RNA genomes. Sequencing of nucleic acid derived from specimens of infected patients were approved by the Institutional Ethics Committee of the National Institute for Infectious Diseases, INMI, “L. Spallanzani” (Issue n. 9/2020).

### Phylogenetic analysis and recombination detection

The phylogenetic analysis was carried out comparing the full-length RNA sequence of the E-11 described here, ECHO11_INMI (Accession n: KX527626.1) with 121 Enterovirus prototype sequences retrieved from the Picornavirus homepage [18], and CV-B1 sequence (Accession n: MG845887.1); the accession numbers of whole sequences are provided in Additional file 2. Three different alignment datasets were analyzed separately to highlight the phylogenetic relationships: one of the full-length genome sequences; one of the portion coding for the P1 region of the polyprotein; and the last one of the P3 region of the polyprotein. The phylogenetic tree of each alignment dataset was inferred using the Neighbor-Joining method [20], with 1000 bootstrap replicates. The rate variation among sites was modeled with a gamma distribution (shape parameter = 1). All ambiguous positions were removed for each sequence pair (pairwise deletion option). There were a total of 7620 positions in the final dataset of full genome, 2598 positions in the final dataset of P1, 345 positions in the final dataset of P3. Evolutionary analysis was performed by using MEGA X [21].

The recombination analysis was carried out by using the software Recombination Detection Program version 4 (RDP4) [22]. To identify the best sequences for the recombination detection analysis, we selected non redundant sequences between the prototype reported on the specific Picornaviridae database (http://www.picornaviridae.com/). The final data set was aligned using the MUSCLE program [23] and the alignment was manually controlled. Moreover, the closest specific sequences were added, these are identified by BLAST tool on genome, blasting the sliding windows of 2 Kb with a step of 2 kb.

RDP4 software can identify the specific positions of recombination breakpoint by automatically using multiple methods of analysis (GENECONV [24], CHIMAERA [25], Maximum Chi Square [26], BOOTSCANning [27], Sister Scanning [28], the 3SEQ [29], VisRD [30] and the BURT methods. In RDP4, we chose to check every possible point of recombination along the E11 genome, viewing a window size of 100 nt and using default parameters.

### Specific amplification

To investigate if the infection was caused by a single recombinant enterovirus variant or by a coinfection of two different serotypes, we designed two sets of primers mapping E-11 and Coxsackiesvirus of B species serotypes, upstream and downstream of the recombination site respectively. The sequences of the primers and the position are reported in Table 2.

**Table 2:** Primer sets specific for E-11 serotypes and CV-B serotypes mapped in regions upstream and downstream from recombination breakpoint

Primer name	Sequence	Position (bp)
E11 2F	CCGTTTGTGTCCCTRGATTACTC	1607–1630*
CVB 2F	TTTGCNCCRCTGAGTTATAGCA	1741–1769^#^
E-11 2R	GAATGCGGAARATGTCCAT	1917–1936*
CVB 2R	ANACYTGGGARCCRTTNGTTGA	1965–1986^#^
E11 3F	GTYGGNTGAYCCNGATGTG	6593–6613*
CVB 3F	GTYGGNTGYGAYCCNGACCT	6593–6612^#^
E11 3R	RGTCAARCARCGCCCRACGGGC	7254–7275*
CVB 3R	GGGTCAARCAGCGCCCNACTGG	7028–7049^#^

*Position for E-11 primers are referred to Human echovirus 11 prototype strain Kust/86 (Accession N GenBank: AY167105.1); ^#^CVB primers position are referred to Coxsackievirus B1 prototype sequence (Accession N GenBank: M16560.1)

The E-11 primers were designed comparing eleven full genome sequences of E-11 retrieved from GenBank. The CV-B specific primers were designed, using an alignment of 17 CV-B sequences retrieved from GenBank.

## Results

### Enterovirus isolation

Three isolates were obtained from the clinical samples of the patients involved in the small outbreak. One was obtained from the hepatic biopsy of patient 1, here referred to as Iso_pt1_L, while the others were obtained from the serums of patient 2 and patient 3, named Iso_pt2_S and Iso_pt3_S respectively (see Table 3).

**Table 3:** Legend of analyzed samples

	Origin of isolates/sample types	Sample name
Isolates	From liver biopsy of patient 1	Iso_pt1_L
	From serum of patient 2	Iso_pt2_S
	From serum of patient 3	Iso_pt3_S
Clinical samples	Liver biopsy of patient 1	pt1_L
	Serum of patient 1	pt1_S
	Liver biopsy of patient 0	Pt0_L

### VP1 and full genome sequence analysis

The Enterovirus strains involved in this small outbreak were typed, basing on VP1 partial sequences and all resulted belonging to E-11. Moreover, the phylogenetic analysis performed comparing the VP1 sequences with a set of E-11 strains retrieved from GenBank shows that our strains strictly correlated and segregated in a unique and well separated clade which belongs to the D5 genotype of E-11 according to Li et al. [31], thus confirming the epidemiological link between the Enterovirus infections described here. (Additional file 3).

To further characterize the RNA genome, the EVs isolated in cell culture (Iso_pt1_L, Iso_pt2_S and Iso_pt3_S) and the virus from the liver biopsy of patient 1 (Pt1_L) were entirely sequenced. All nucleotide sequences were then aligned using Clustal W and the amino acid sequences of the polyproteins were compared. As expected, all viral strains revealed a high identity at nucleotide level (median = 99.87%, ranging from 99.83 to 100%); two schematic tables of the nucleotides and amino acid differences respectively found, are provided in Additional file 4. Moreover, comparing the Liver Enterovirus strain (Pt1_L) with the corresponding Liver isolate (Iso_pt1_L), no amino acid differences were observed, while the sequence of Iso_pt2_S compared to Enterovirus strains of pt1, shows one amino acid substitution, C1677 L, located in the P3 region of the polyprotein. Specifically, it maps in the region encoding for the protein complex of 3BCD that is the precursor of the non-structural proteins 3B (Vpg primers for RNA transcription), 3C (protease) and 3D (viral polymerase). Similarly, we compared the amino acid sequence of Iso_pt3_S with those of pt1 (Iso_pt1_L and pt1_L) and we found the following four substitutions: D478G localized in the P1 portion of the polyprotein encoding for capsid proteins; T1898A, G2100V and E2101T, located in the portion encoding for the viral RNA polymerase 3D. Furthermore, comparing the amino acid sequence of the virus described here with 35 polyprotein sequences of other E-11 strains downloaded from Genbank, we found 25 amino acid substitutions (V101I, E115D, I310V, V572A, T651V, E895D, Q1031H, C1033S, L1938F, I1084V, T1122A, Q1185H, S1213E, S1365N, T1418S, S1429N, N1536S, P1539L, A1533S, V1558I, L1298I, T1868N, I2135V and D2140N). To date, no biological significance associated with any of these substitutions have been described.

**Fig. 2:**
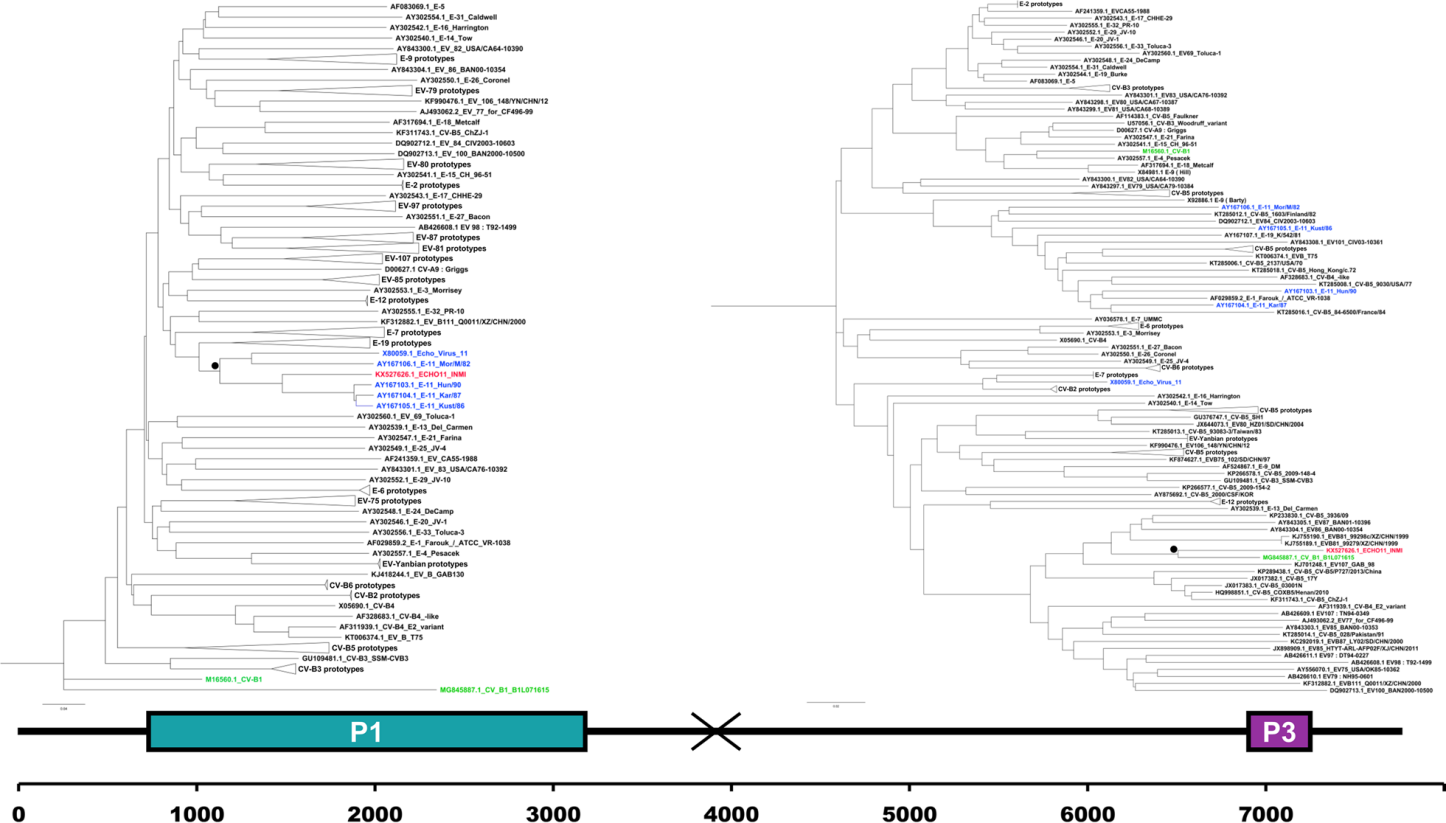
Phylogenetic trees. Phylogenetic trees constructed on the basis of P1 region [nucleotide positions from 726 to 3253 referring to Human echovirus 11 prototype strain Kust/86 (Accession N GenBank: AY167105.1)] and of the P3 region [nucleotide positions from 6956 to 7256 referring to Human echovirus 11 prototype strain Kust/86 (Accession N GenBank: AY167105.1)]. The nodes defining the clade including ECHO11_INMI strain are indicated with a black dot (Bootstrap value in a, b, and c tree are: 99, 100 and 94 respectively). ECHO11_INMI strain are reported in red, Echo 11 types are reported in blu and CVB1 types are reported in green

Despite the typing of the VP1 gene assigned our virus to an E-11, the phylogenetic analysis of the full-genome sequence of ECHO11_INMI against a set of 123 sequences retrieved from the Picornavirus homepage, showed an unexpected pattern: ECHO11_INMI strain did not segregate with E-11 serotypes, but with a CV-B1 (Acc N: MG845887) (Fig. 2). This finding led us to hypothesize that ECHO11_INMI could be a chimeric strain, maybe originating from a recombination event between an E-11 (Acc N: AY167103) and CV-B1 (Acc N: MG845887).

Unfortunately, we were not able to sequence the entire genome of the virus infecting Pt 0 (index case) due to the low quantity of virus in residual material, as the liver bioptic sample (Pt0_L) was fixed in paraffin which badly conserved the nucleic acid. However, we were able to obtain two amplicons of the sequence by RT-PCR that localized upstream and downstream from the recombination breakpoint respectively. Both amplicons were sequenced and show a high identity (median value = 99.78% and 98.17% of upstream and downstream fragment, respectively) both with the sequences of the three isolates and the virus detected in the liver of patient 1 (Pt1_L), therefore suggesting that the recombination had already occurred in the virus infecting the source (Pt0).

### Phylogenetic analysis

The phylogenetic tree of the full genome sequences (data not shown) shows that ECHO11_INMI, segregates with the CV-B1 (MG845887.1). To better define the phylogenetic relationships, we analyzed separately the P1, containing the VP1 gene, and the P3 region as it locates close to 3′ end of the genome and far from P1 (Fig. 1). The phylogenetic tree of the P1 region (Fig. 2) shows that our ECHO11_INMI sequence clusters together with all E-11 types while CV-B1 (MG845887.1) segregates with CV-B1 type in a separate clade. The P1 region contains the sequence coding for the capsid proteins, VP4, VP2, VP3 and VP1. In particular, it is well known that VP1 is the most antigenic protein and its sequence is used for the typing of Enterovirus genus as it has been shown to correlate very well with the classical serotype [32]. Indeed, phylogenetic studies on VP1 sequences of the genus have clearly shown that strains of the same serotype always cluster together [33].

Therefore, this result confirms that our sequence belongs to the E-11 type. The last tree (Fig. 2), constructed on the basis of the P3 region of the genome, shows that the ECHO11_INMI segregates close to the CV-B1 (MG845887.1). This result is consistent with the hypothesis that recombination occurred in the P2 region of the genome, between P1 and P3.

### Recombination plot

**Fig. 3:**
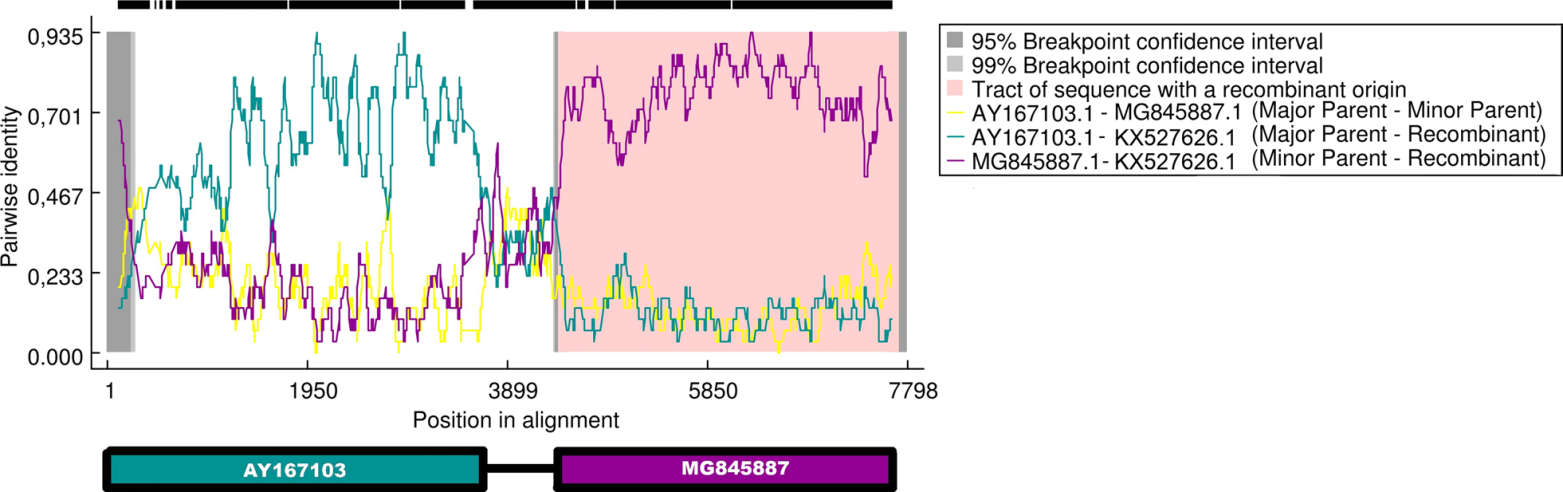
Recombination analysis results using RDP4. The plot shows the pairwise identity between ECHO11_INMI (accession no: KX527626) and the Human E-11 strain Hun/90 (accession no: AY167103.1) or CV-B1 (accession n. MG845887), represented in green and purple line respectively. While the yellow line shows the pairwise identity between E-11 strain Hun/90 and CV-B1. Each polymorphic site detected in the sequence dataset analyzed, was marked with a black bar. Then, the long black lines above the plot equivalent to the high number of nucleotide variation among sequences; the white spaces identify regions where the polymorphism are absent

To confirm the presence of a recombination breakpoint in our strains, we performed a recombination detection analysis, using RDP4 software (Fig. 3). Specifically, the analysis recognized that ECHO11_INMI was a chimeric strain of E-11 (AY167103) and CV-B1 (MG845887); it also identified the breakpoint of recombination between nucleotide 4083 and 4201 of ECHO11_INMI sequence without gap, with 99% certainty (p values 5.259*10^−24^, as reported by RDP4).

The recombination site is located in the region encoding for P2 of the polyprotein that is the precursor of three non-structural proteins involved in the replication process: 2Apro, 2B and 2C.

### Specific amplification

To confirm that the virus in our samples is a new variant originating from a recombination event between an E-11 and a CV-B1, and to exclude the hypothesis of a co-infection with both viruses, we designed four sets of primers. Two of them were specific for E-11 serotype, targeting respectively the region upstream and downstream of the breakpoint (E11 2F-E11 2R, E11 3F-E11 3R); in the same way we designed two sets of primers specific for CV-B serotypes, targeting respectively the region upstream and downstream from the recombination site (CVB 2F-CVB 2R, CVB 3F-CVB 3R). Figure 1 shows a schematic representation of the EVs genome and details of experimental design for RT-PCR amplification of both E-11 and a CV-B1.

**Fig. 4:**
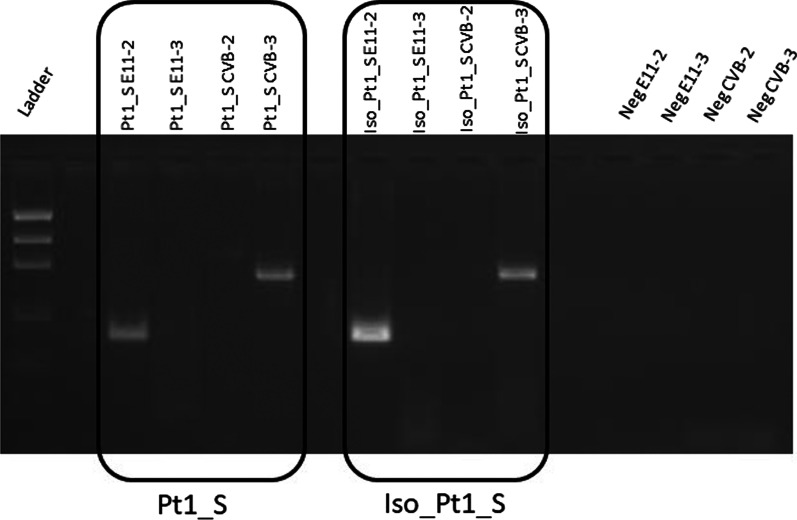
RT-PCR performed with specific sets of primers for E-11 and CV-B on Pt1_S and Iso_PT1_S

Pt1_S and all isolates were tested with all sets of primers described and we obtained similar results (Additional File 5). The amplification resulted positive with the set E112F-E112R, that targets the region upstream of the recombination breakpoint, and with the set CVB 3F-CVB 3R, that targets the region downstream of the breakpoint, instead the other sets of primers, CVB 2F-CVB 2R and E11 3F-E11 3R, that map at 5′ and 3′ of the genome, respectively, gave negative results (Fig. 4).

These findings are consistent with the recombination hypothesis and confirm the presence of only the recombinant variant in the examined samples; in addition the presence of the same pattern of amplification obtained by the analysis of the virus in serum sample and the one isolated from cell culture, revealed that the recombination was not generated by the isolation procedure.

## Discussion

The genome of Enteroviruses evolves rapidly due to the action of two main evolutionary mechanisms: the high accumulation of point mutations caused by the activity of the error-prone RNA-dependent RNA-polymerase lacking a proofreading activity, as well as the property of EVs to undergo recombination of the RNA genome easily.

The first mechanism mainly contributes to an increment of genetic drift due to the high frequency of nucleotide misincorporation (generating a quasi-species population). On other hand, the second leads to a much more extensive genetic variation, due to the exchange of a large portion of the genome among distinct RNA strands that determines simultaneous insertion of multiple substitutions in a single new genome.

The recombination process does not create new mutations in the existing genetic repertoire of the virus, but creates new combinations of pre-existing polymorphisms. Therefore, such a mechanism has been considered the major driving force in Enterovirus evolution, allowing the virus to rapidly explore a larger sequence space than the slow accumulation of point mutations.

Both mutation mechanisms are crucial for viral adaptability, escape from the host immune response, dissemination and pathogenesis but recombination is the one that mainly contributes to the emergence of new strains with much more burden potential [34, 35].

In this study, we have reported the characterization of a recombinant E-11 associated with a small nosocomial outbreak with fatal outcome. The molecular diagnostic of cases revealed an Enterovirus infection. Despite the VP1 typing having initially assigned the strain to E-11, the analysis of the full-genome sequence revealed that the virus was instead generated by a recombination event which occurred between an E-11 and a CV-B1. Indeed, phylogenetic analysis carried out separately on P1 and P3 of the polyprotein region show two different phylogenetic patterns: the P1 of our strain closely related to E-11 types while the P3 cluster together with CV-B1 types.

This phylogenetic discrepancy observed, led us to hypothesize that a recombination event had occurred between an E-11 and a CV-B1 thus, generating such strain. To investigate this recombination hypothesis more in depth, we performed a recombination detection analysis, using RDP4 software, which stated that ECHO11_INMI was a recombinant and the breakpoint was located in the P2 region of the genome. The P2 region is considered a recombination hotspot for enterovirus genome and frequently breakpoints are located in this portion of the polyprotein [36].

Finally, we designed two sets of primers specific for E-11 and two sets specific for CV-B1, targeting the region upstream and downstream from the recombination breakpoint. The PCR performed with these primers stated that only the recombinant virus was present in the clinical samples, excluding the simultaneous presence of both Enterovirus types and strengthening the hypothesis of recombination.

Both E-11 and CV-B1 belong to the B species of the Enterovirus genus that are often associated with neonatal infections and in particular with aseptic meningitis; in addition, E-11 is frequently associated with acute flaccid myelitis [37], while CV-B1 frequently causes myocarditis, sepsis, and hepatitis, which can rapidly deteriorate to critical status [38].

Echovirus E-11 is one of the most commonly isolated enterovirus serotypes [39]. It generally causes mild disease in immunocompetent adults, however, the infection may progress to severe presentation in infants or immunocompromised patients [40], as in the case of Pt 0 of this nosocomial cluster. The reason why one of the healthcare workers who took part in the autopsy developed a severe acute syndrome, rarely reported in immunocompetent patients, and died from liver failure, is unclear. As far as it may be hypothesized, this occurred due to a massive exposure to contaminated material during autopsy, eventually resulting in a hyper-acute infection. But a role played by the chimeric structure of the viral genome may not be excluded; further biological characterizations are necessary to highlight the pathogenetic implications of the recombinant E-11 viral genome described here.

Recombination is an event frequently reported for Enteroviruses that occur in co-infected cells, especially among the same Enterovirus species and recombination events have been frequently described for E-11 serotypes [41]. Two mechanisms responsible for these events have been described: the replicative mechanism of copy-choice and the non-replicative mechanism. The former is considered responsible for the highest number of recombination events in RNA viruses [36].

Such a condition could make Enteroviruses, which have a highly variable genome due to the lack of proofreading activity of RNA-dependent RNA polymerase, even more prone to mutating and transforming.

## Conclusions

Recombination events could lead to the sudden emergence of new Enterovirus variants with a high pathogenic power that frequently manifest themselves in the form of epidemic transmission. This study highlights the importance of carrying out surveillance activities of Enteroviruses not only monitoring the genotypes defined on the partial portion of theVP1 gene, but deeply characterizing the entire genome of circulating Enterovirus strains. The typing techniques routinely in use to date, based on the VP1 gene, provide only partial information and the occurrence of emerging recombinant strains with a higher pathogenic potential may be underestimated.

## Supplementary Information

**Additional file 1**. Sequences of primers used for full genome sequences obtainment.

**Additional file 2**. Sequences used for Phylogenetic analysis. Lists of genome sequence used for phylogenesis analysis for Full Genome, P1 and P3 trees.

**Additional file 3**. Phylogenetic analysis on the basis of VP1 sequences.

**Additional file 4**. Comparison of nucleotide and amino acid sequences. Sequence comparison of nucleotide and amino acid of all full-length sequences.

**Additional file 5**. Specific amplification. Electrophoresis gel of Pt2 and Pt3 serum samples.

## Data Availability

The sequences of ECHO11_INMI have been deposited in GenBank with Accession Number: Iso Pt1_L_FullGenome: KX527626. Iso_Pt2_S_FullGenome: MZ597838. Iso_Pt3_S_FullGenome: MZ597839. Pt1_L_FullGenome: MZ597840. Pt0_L_VP1: MK388908.1 Pt1_L_VP1: MK388904.1 Iso_Pt1_S_VP1: MK388905.1 Iso_Pt2_S_VP1: MK388906.1 Iso_Pt3_S_VP1: MK388907.1

## Abbreviations

E-11: Echovirus 11
CV-B: Coxsackievirus B
CV-B1: Coxsackievirus B1
EVs: Enteroviruses
ORF: Open reading frame
UTR: Untranslated region
IRES: Internal ribosome entry site
HFMD: Hand, foot and mouth disease
AFP: Acute flaccid paralysis
MEM: Modified eagle medium
FCS: Fetal calf serum
RDP4: Recombination Detection Program 4
