# Supplementary figures and images for: Detection of recombinant breakpoint in the genome of human enterovirus E11 strain associated with a fatal nosocomial outbreak
Source: Virol J. 2022 Jun 3;19:97. doi: 10.1186/s12985-022-01821-2 (PMC9166486; doi:10.1186/s12985-022-01821-2)

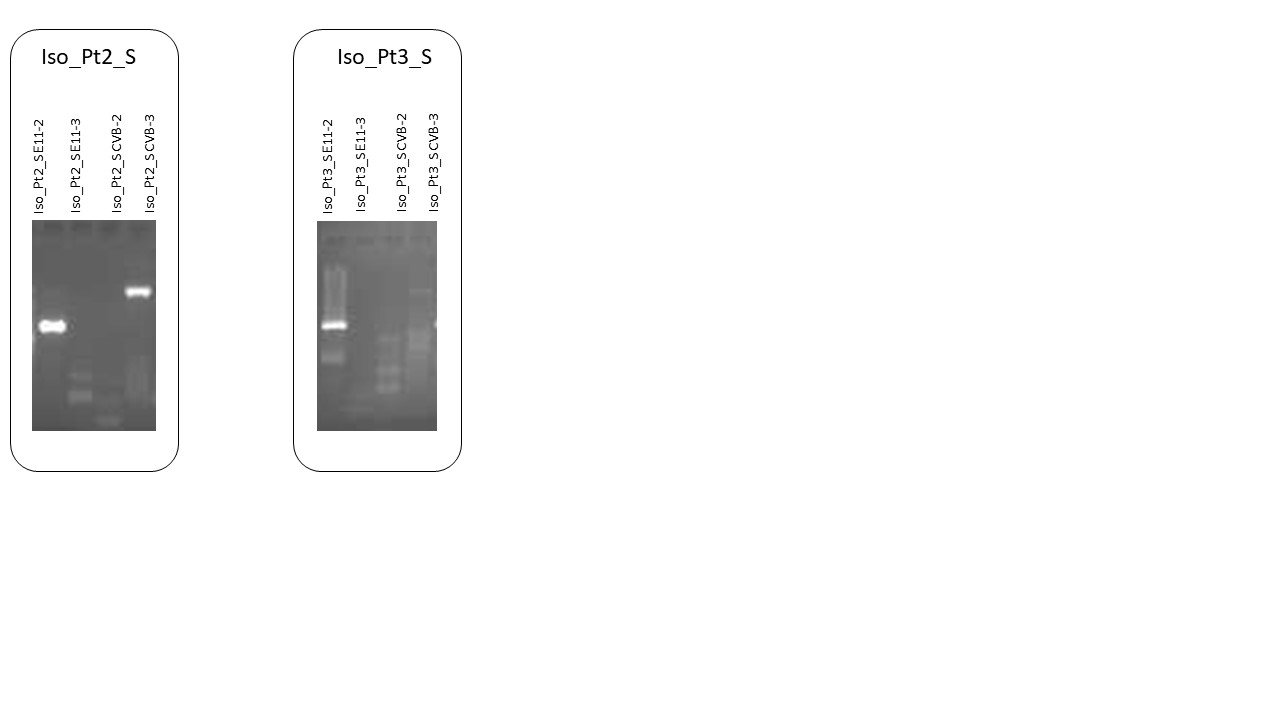

Supplement: Supplementary file 5 — Additional file 5. Specific amplification. Electrophoresis gel of Pt2 and Pt3 serum samples. [file 12985_2022_1821_MOESM5_ESM.jpg]
